# Deep-learning-based buffalo identification through muzzle pattern images

**DOI:** 10.5194/aab-68-473-2025

**Published:** 2025-07-14

**Authors:** Orhan Ermetin, Humar Kahramanlı Örnek

**Affiliations:** 1 Faculty of Agriculture, Department of Animal Science, Yozgat Bozok University, Yozgat, Türkiye; 2 Department of Computer Engineering, Selcuk University, Konya, Türkiye

## Abstract

The rapid advancement of artificial intelligence systems has accelerated applications across various fields, including animal biometrics. Accurate identification of buffaloes is crucial for producers and researchers to maintain records and ensure effective tracking. In this study, artificial-intelligence-supported buffalo recognition was developed as an identification method for large livestock. Facial images of 11 buffalos from a facility in the province of Yozgat were utilised to create a dataset for the study. All four algorithms demonstrated successful results. Notably, SqueezeNet outperformed the others, with a remarkable 99.88 % accuracy, 0.998 precision, 0.999 recall, and an F1 score of 0.999. Besides this, ResNet101 was the least successful method, with 99.30 % accuracy, 0.979 precision, 0.995 recall, and an F1 score of 0.987. The accuracy of SqueezeNet and GoogLeNet is 99.88 %, and the recall of these algorithms is 0.999. The precision of SqueezeNet is 0.998, while GoogLeNet's precision is 0.997. The F1 scores of SqueezeNet and GoogLeNet are 0.999 and 0.998, respectively.

## 1 Introduction

Livestock farming is changing from small-scale farming to intensive and specialised breeding. Complex factors such as labour shortages, real-time monitoring difficulties, and high management costs have a challenging impact on large-scale production systems that require the use of technology (Xu et al., 2021). Utilising technology in animal husbandry enhances efficiency and provides various benefits, such as health monitoring, feed management, and eco-friendly practices. These innovations enable producers to achieve sustainable and profitable operations through early detection, data analysis, and efficient communication tools. Identification, verification, and monitoring of farm animals are important for controlling animal movements and preventing diseases (Awad, 2016). Marking animals using traditional methods can negatively affect the animals' behaviour and have harmful consequences. This may result in erroneous data in research results (Mori et al., 2000). Metal ear tags; flexible plastic ear tags (Washington, 1995); and, more recently, subcutaneous RFID (radio frequency identification) tags (Voulodimos et al., 2010) and rumen boluses are used extensively for animal identification. Ear tags worn in the ear are difficult to read and may fall off or be replaced. Using subcutaneous injected RFIDs or rumen boluses requires expertise. In their application, serious accidents may cause animal injuries or even death (Bugge et al., 2011; Lu et al., 2014). New searches and studies are being conducted to reduce the problems and deadlocks encountered in traditional identification methods. For this purpose, biometric identification methods have begun to be used in addition to existing animal identification methods.

With the spread of artificial intelligence, deep learning, artificial neural networks, and automation technologies, these modern technologies in traditional animal husbandry practices have gained importance in recent years (Saleem et al., 2021). Adopting these technologies will significantly reduce human labour, increase efficiency in modern production, and improve product quality (Neethirajan and Kemp, 2021). Visual animal biometrics is a research field combining computer vision, pattern recognition, and cognitive science, which also plays a role in the analysis of animal behaviour (Kühl and Burghardt, 2013). Various biometric features are used to define the unique identities of animals (Dandıl et al., 2019). Features such as muzzle print patterns, iris patterns, retinal vascular patterns, facial images, and DNA profiles are the unique characteristics of each animal, and, by taking advantage of these characteristics, it is possible to distinguish animals through a process called biometric identification (Jiménez-Gamero et al., 2006; Rojas-Olivares et al., 2012). Biometric methods provide high security while maintaining accuracy and reliability by automating authentication and identification systems (Awad, 2016).

In animals, muzzle prints are biometric identifiers, like fingerprints in humans, and are unique to each individual (Minagawa et al., 2002). Distinguishing animals based on muzzle prints is a subject that has been studied for many years (Baranov et al., 1993). Animal muzzle prints can be made using ink printing or digital painting. Moisture accumulation in the animal's muzzle and the inability to keep it still lead to smeared and unreadable images, making it a problematic and time-consuming method to apply (Barry et al., 2007). Using digital images gives easier and faster results. Minagawa et al. (2002) studied this method and used combination pixels on depth traces for muzzle print matching. The researchers identified 30 animals with an overall accuracy rate of 66.6 %. However, the results are unsatisfactory due to low-quality trace images, a limited database size, and limited performance of feature extraction and matching learning algorithms. Conducting a similar study, Noviyanto and Arymurthy (2012) applied the speeded-up robust features (SURF) approach to muzzle print images for cattle identification. A total of 120 muzzle print images from eight animals were obtained with the U-SURF method, and 90 % maximum identification accuracy was achieved by using 10 images in training and 5 images in input samples. Awad et al. (2013) gained 93.3 % identification accuracy based on 105 images from 15 cattle using a previously collected database. Noviyanto and Arymurthy (2013) achieved an accuracy of 90.6 % in identifying cattle by applying the SURF model to a dataset of 80 images. Gaber et al. (2016) used the AdaBoost classifier on a database of 31 cattle. They achieved 99.5 % classification accuracy. El-Henawy et al. (2016) applied the J48 decision tree and naïve Bayes methods to classify animals and stated that the decision tree classifier gave more accurate results than the naïve Bayes classifier. Andrew et al. (2019) used YOLOv2 based on a deep convolution network and CNNs (convolutional neural networks) to classify images from videos with 94.4 % accuracy.

Bello et al. (2020) obtained 98.99 % accuracy in 4000 images by pre-processing, Gaussian filtering, and deep learning using the images they collected using the muzzle database and the stacked deep learning technique. Shojaeipour et al. (2021) achieved 99.11 % accuracy in detection by muzzle by applying the two-stage YOLOv3–ResNet50 algorithm to images of cattle's muzzle tissues.

Water buffaloes are disease-resistant and contented animals that can adapt to various environmental conditions and from which people have benefited from various products such as meat, milk, and leather for centuries (Şahin et al., 2013). Regarding milk production, water buffalo are the second most important species worldwide after dairy cows and produce high-quality milk (Ermetin, 2017). Research on water buffalo breeding and adaptation to precision agriculture technologies (PLF technologies) is limited (Kul et al., 2018; Ermetin, 2021). Due to the aggressive nature of water buffaloes, it is difficult to approach them, to read their ear tags, or to interfere with them; the intervention of a stranger, especially, may cause injuries (Ermetin, 2023). The fact that existing ear tags cannot be read and fall off makes it important to overcome these problems by identifying them with artificial intelligence. The muzzle recognition method makes identifying animals without approaching them easier, allowing for the quick identification of their identity and necessary interventions.

Van Steenkiste et al. (2023) developed a deep-learning model to detect such clots on pictures of the milk filter socks of the milking system. In total, 1282 pictures were taken, with 696 and 586 of them being with and without clots, respectively. CNNs (convolutional neural networks) with residual connections were trained. Hyperparameters were optimised using a genetic algorithm. They achieved 100 % classification accuracy. El Hadad et al. (2015) used two models, namely ANNs (artificial neural networks) and kNN (K-nearest-neighbour classifier), to classify bovines. They collected images from 28 bovine animals. ANNs achieved 92.76 % classification accuracy, while kNN achieved 100 % classification accuracy. Kumar et al. (2018) proposed the deep-learning-based approach to identification of individual cattle based on their muzzle pattern images to address the problem of missed or swapped animals and false insurance claims. Convolution neural networks were used for cattle recognition. A total of 98.99 % identification accuracy was achieved.

Madkour and Abdelsabour-Khalaf (2022) presented a method for identifying animal species using scanning electron microscopic studies of nasal skin. They examined the nasal skin of seven animal species using scanning electron microscopy (SEM). They stated that the skin around the nostrils plays an important role as a means of identification in forensic investigations and improves the field of veterinary forensic medicine in general.

Our research aimed to recognise water buffaloes based on their muzzle pattern image by using artificial intelligence techniques in water buffalo breeding, where precision farming techniques are not applied in terms of temperament and breeding methods; it was also our aim to have this research be included in the literature on this method.

**Figure 1:**
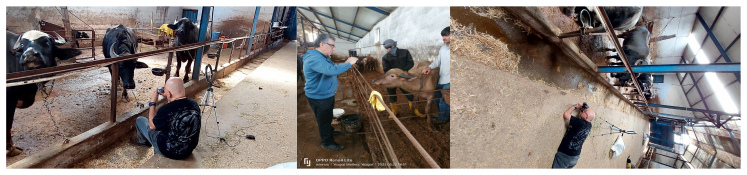
Scenes from the process of taking buffalo images. Photo by Orhan Ermetin.

Deep learning, which is the field of artificial intelligence, has made significant strides to solve problems that have resisted the best efforts of the artificial intelligence community for many years. It has proven to be very good at detecting complex structures in high-dimensional data and is therefore applicable to many areas of science, business, and government (LeCun et al., 2015). Convolutional neural networks (CNNs), which are deep neural networks (DNNs), are designed to process data that come in the form of multiple arrays, such as a colour images consisting of three 2D arrays containing pixel intensities in three colour channels (LeCun et al., 2015). It was modelled based on the observation of biological processes and mimics the functions of different layers of the human brain (Shanthi and Sabeenian, 2019). Convolutional neural network (CNN) models with self-learning abilities and superior classification results on multi-class problems could achieve successful accuracy in image classification problems. CNNs consist of a chain of convolution layers, pooling layers, and batch normalisation operations.

There are several well-known architectures of CNNs:

- LeNet is one of the earliest CNN structures proposed by LeCun et al. (1998).
- AlexNet was proposed in 2012 by Krizhevsky et al. (2017) and became known after competing in the ImageNet Large Scale Visual Recognition Challenge (ILSVRC).
- ZF Net is an improvement on AlexNet, presented by Zeiler and Fergus (2014). The method won ILSVRC 2013.
- GoogLeNet (or Inception V1) was proposed by researchers at Google in 2014 (Szegedy et al., 2015). This method won ILSVRC 2014.
- VGGNet was proposed by Simonyan and Zisserman (2014). The algorithm was one of the most popular models submitted to ILSVRC 2014.
- Residual Neural Network (ResNet) was developed by He et al. (2016) and won the ILSVRC 2015.

This research employs four CNN-based algorithms, namely AlexNet, SqueezeNet, GoogLeNet, and ResNet101.

## 2 Materials and method

### 2.1 Material

In this study, images were captured of 11 buffaloes on a water buffalo farm located in the central district of the province of Yozgat. A dataset was compiled by recording facial photographs and other images of the water buffaloes at various time intervals using a camera. Images were collected from 40 healthy buffaloes of different ages; the age distribution was taken into account during the study. The images were recorded from a suitable distance, with the buffalo's face being visible from different angles (Fig. 1). There are a total of 4263 images from 11 buffalo in this dataset. The images have RGB colour spacing. The images of the buffaloes obtained throughout the study were numbered 1–11.

CNNs have different architectures like LeNet, AlexNet, GoogLeNet, ConvNet, and ResNet. This study employs four models of them, namely AlexNet, SqueezeNet, GoogLeNet, and Resnet101. The methods used will be introduced in this section.

### 2.2 AlexNet

**Figure 2:**
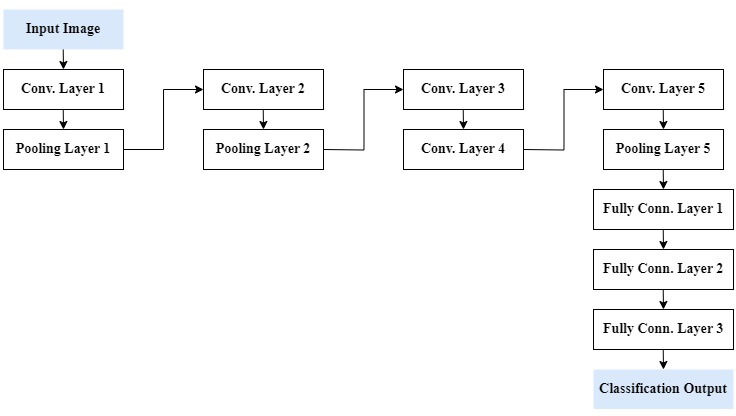
The structure of AlexNet.

AlexNet, which belongs to a deep CNN structure, was proposed by Krizhevsky et al. (2017). AlexNet achieved high classification accuracy for the ImageNet dataset, a significant breakthrough in the field of machine learning. Since then, researchers have begun to devote more time and effort to researching deep learning models (Lu et al., 2021). AlexNet consists of five convolutional and three full-connection layers. After the first, second, and fifth convolutional layers, maximum pooling is performed. The input format of the source data is 227×227×3 pixels, where 227 pixels represent the width and height of the input image, and 3 pixels represent a three-channel RGB mode of the data source (Chen et al., 2021). The structure of AlexNet is shown in Fig. 2, and the detailed architecture is introduced in Table 1.

**Table 1:** The architecture of AlexNet.

Layer	Layer type	Layer details
1	Image input	227×227×3 images
2	Convolution	96 11×11×3 convolutions with stride [4, 4] and padding [0, 0, 0, 0]
3	ReLU	ReLU
4	Cross-channel normalisation	Cross-channel normalisation with five channels per element
5	Max pooling	3×3 max pooling with stride [2, 2] and padding [0, 0, 0, 0]
6	Grouped convolution	Two groups of 128 5×5×48 convolutions with stride [1, 1] and padding [2, 2, 2, 2]
7	ReLU	ReLU
8	Cross-channel normalisation	Cross-channel normalisation with five channels per element
9	Max Pooling	3×3 max pooling with stride [2, 2] and padding [0, 0, 0, 0]
10	Convolution	384 3×3×256 convolutions with stride [1, 1] and padding [1, 1, 1, 1]
11	ReLU	ReLU
12	Grouped convolution	Two groups of 192 3×3×192 convolutions with stride [1, 1] and padding [1, 1, 1, 1]
13	ReLU	ReLU
14	Grouped convolution	Two groups of 128 3×3×192 convolutions with stride [1, 1] and padding [1, 1, 1, 1]
15	ReLU	ReLU
16	Max Pooling	3×3 max pooling with stride [2, 2] and padding [0, 0, 0, 0]
17	Fully connected	4096 fully connected layers
18	ReLU	ReLU
19	Dropout	50 % dropout
20	Fully connected	4096 fully connected layers
21	ReLU	ReLU
22	Dropout	50 % dropout
23	Fully connected	1000 fully connected layer
24	Softmax	Softmax
25	Classification output	crossentropyex

### 2.3 SqueezeNet

**Figure 3:**
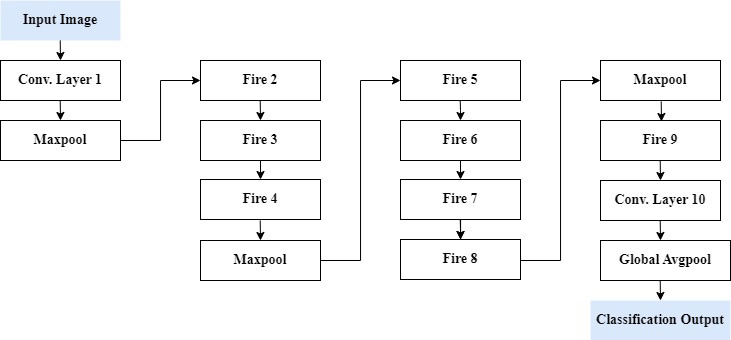
The structure of SqueezeNet.

SqueezeNet is a type of CNN that aims to achieve improved efficiency compared to AlexNet by using 50 times fewer parameters. It consists of a total of 15 layers, including three max-pooling layers, two convolutional layers, eight fire layers, one softmax output layer, and one global average pooling layer (Minu et al., 2022). The layered framework of the network is introduced in Fig. 3.

### 2.4 GoogLeNet

**Figure 4:**
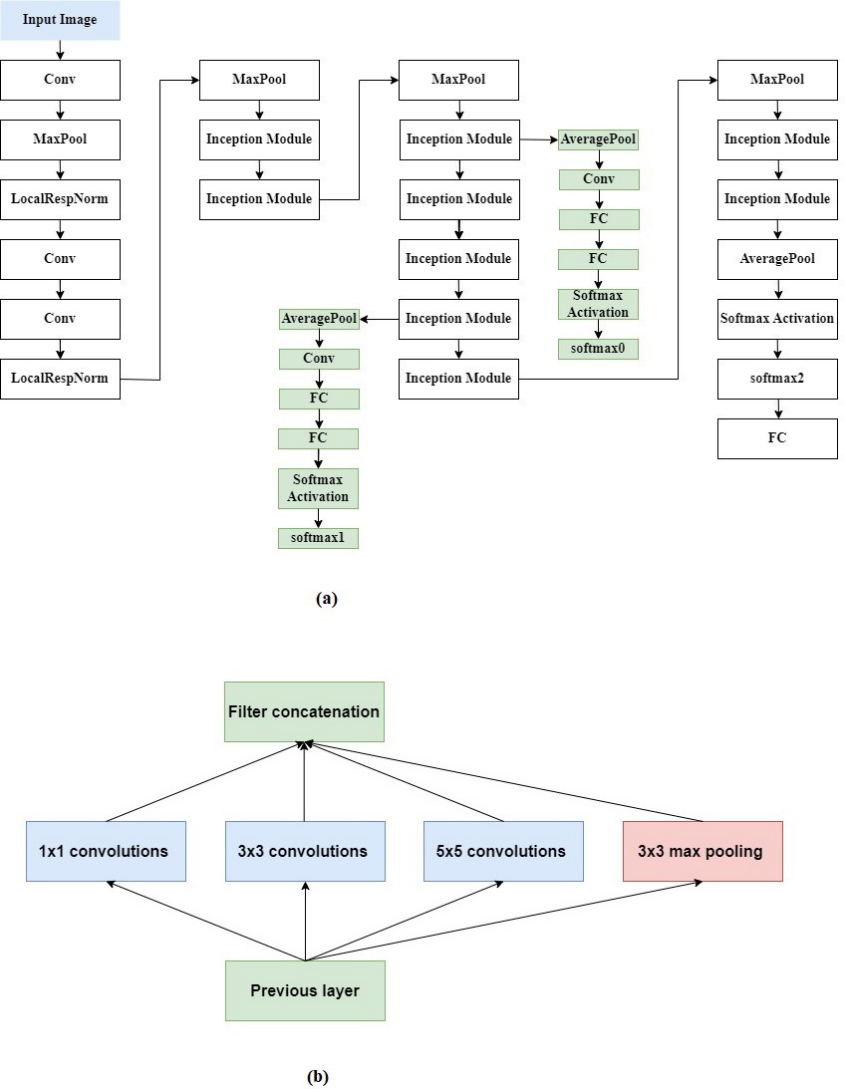
**(a)** The structure of GoogLeNet. **(b)** Inception module.

GoogLeNet (Szegedy et al., 2015), also known as Inception, is a popular deep CNN architecture with 22 layers developed to outperform the existing CNN architectures. GoogLeNet is notable for its architecture, which employs Inception modules (IMs). These modules combine filters at different scales to enhance the model's ability to extract information. Additionally, the model aims to reduce the number of parameters and to increase computational efficiency by incorporating various techniques. IM allows multiple convolutions with different kernels and simultaneous max pooling, ensuring that the network trains with optimal weights and identifies more meaningful features (Balagourouchetty et al., 2019). The structure of GoogLeNet and the Inception module is shown in Fig. 4.

### 2.5 ResNet

**Figure 5:**
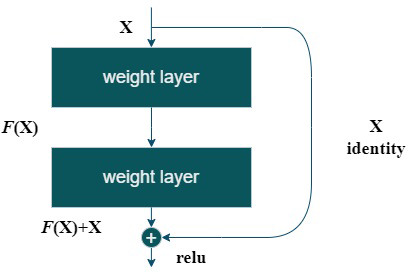
ResNet shortcut connections.

He et al. (2016) introduced a residual block containing two convolutional layers and a non-parameterised shortcut connection that passes the previous block's output to the next unmodified block. The residual block is shown in Fig. 5.

### 2.6 Performance metrics

In this study, a confusion matrix and evaluation metrics were employed to measure the success of the classification. Therefore, a complexity matrix for two class problems was created based on the definition of actual values and predicted values in Table 2.

**Table 2:** Confusion matrix definition.

Reference	Actual	
	Class 0	Class 1
Class 0	True positive (TP)	False positive (FP)
Class 1	False negative (FN)	True negative (TN)

Each column of the confusion matrix represents the instances in a predicted class, while each row represents the instances in an actual class (or vice versa). In the context of binary classification, a true positive (TP) is an outcome where the model correctly predicts the positive class. A true negative (TN) is an outcome where the model correctly predicts the negative class. A false positive (FP) is an outcome where the model incorrectly predicts the positive class, and a false negative (FN) is an outcome where the model incorrectly predicts the negative class. TP, TN, FP, and FN results are used together to calculate evaluation metrics such as accuracy, sensitivity, precision, and F1 score.

**Table 3:**
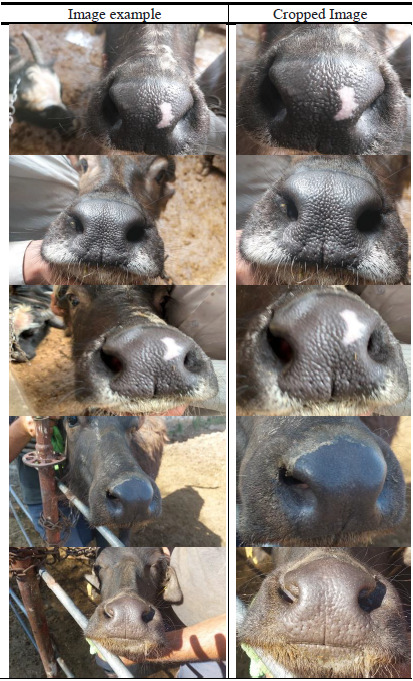
Examples of images of buffaloes and their cropped images.

**Table 4:** Confusion matrix of AlexNet.

		Actual buffaloes										
		Buffalo 1	Buffalo 2	Buffalo 3	Buffalo 4	Buffalo 5	Buffalo 6	Buffalo 7	Buffalo 8	Buffalo 9	Buffalo 10	Buffalo 11
Recognised buffaloes	Buffalo 1	140										
	Buffalo 2		77									
	Buffalo 3			97								
	Buffalo 4				90							
	Buffalo 5					33						
	Buffalo 6						46					
	Buffalo 7							87				
	Buffalo 8								86			
	Buffalo 9									102		
	Buffalo 10									1	20	
	Buffalo 11											73

**Table 5:** Confusion matrix of SqueezeNet.

		Actual buffaloes										
		Buffalo 1	Buffalo 2	Buffalo 3	Buffalo 4	Buffalo 5	Buffalo 6	Buffalo 7	Buffalo 8	Buffalo 9	Buffalo 10	Buffalo 11
Recognised buffaloes	Buffalo 1	140										
	Buffalo 2		77									
	Buffalo 3			97								
	Buffalo 4				90							
	Buffalo 5					33						
	Buffalo 6						46			1		
	Buffalo 7							87				
	Buffalo 8								86			
	Buffalo 9									102		
	Buffalo 10										20	
	Buffalo 11											73

**Table 6:** Confusion matrix of GoogLeNet.

		Actual buffaloes										
		Buffalo 1	Buffalo 2	Buffalo 3	Buffalo 4	Buffalo 5	Buffalo 6	Buffalo 7	Buffalo 8	Buffalo 9	Buffalo 10	Buffalo 11
Recognised buffaloes	Buffalo 1	140										
	Buffalo 2		77									
	Buffalo 3			97								
	Buffalo 4				90							
	Buffalo 5					33		1				
	Buffalo 6						46					
	Buffalo 7							86				
	Buffalo 8								86			
	Buffalo 9									103		
	Buffalo 10										20	
	Buffalo 11											73

**Table 7:** Confusion matrix of ResNet101.

		Actual buffaloes										
		Buffalo 1	Buffalo 2	Buffalo 3	Buffalo 4	Buffalo 5	Buffalo 6	Buffalo 7	Buffalo 8	Buffalo 9	Buffalo 10	Buffalo 11
Recognised buffaloes	Buffalo 1	140										
	Buffalo 2		77									
	Buffalo 3			97								
	Buffalo 4				90							
	Buffalo 5					33						
	Buffalo 6						46					
	Buffalo 7							87				
	Buffalo 8								86			
	Buffalo 9									97		
	Buffalo 10									6	20	
	Buffalo 11											73

The key performance metrics used to evaluate classification performance are as follows:

- *Accuracy*. Accuracy is used to measure a model's performance on classification problems. This metric measures the ratio of samples correctly classified. The formula for accuracy is shown in Eq. (1):
1 $$\text{Accuracy}=\frac{\text{TP}+\text{TN}}{\text{TP}+\text{TN}+\text{FP}+\text{FN}}.$$
- *Precision*. Precision is calculated as the ratio of positive examples classified as positive by the model to positive ones that are actually classified as positive. The formula for precision is shown in Eq. (2):
2 $$\text{Precision}=\frac{\text{TP}}{\text{TP}+\text{FP}}.$$
- *Recall*. Recall is calculated as the ratio of samples that are positive and classified as positive by the model to samples that are actually positive. The formula for recall is shown in Eq. (3):
3 $$\text{Recall}=\frac{\text{TP}}{\text{TP}+\text{FN}}.$$
- *F1 score*. The F1 score is a measure of the harmonic mean of precision and recall. The formula for F1 score is shown in Eq. (4):
4 $$\text{F1 score}=\frac{2\left(\frac{\text{TP}}{\text{TP}+\text{FN}}\right)\left(\frac{\text{TP}}{\text{TP}+\text{FP}}\right)}{\left(\frac{\text{TP}}{\text{TP}+\text{FN}}\right)+\left(\frac{\text{TP}}{\text{TP}+\text{FP}}\right)}.$$

## 3 Results and discussion

In the application stage, the first pre-processing was done. The unwanted areas on the images were cropped. ACDSee Pro software was used for this aim. Table 3 presents sample images and cropped images of some buffalos of the dataset.

The dataset was divided into training and testing sets randomly. The training set contains 80 % of samples. The remaining 20 % of samples are used for testing. Thus, training and testing sets contain 3411 and 852 samples, respectively. The models were implemented in MATLAB R2023b with Intel Core i7 7700HQ CPU, 16 GB RAM, and 8 GB Nvidia GeForce GTX 1070 GPU.

**Figure 6:**
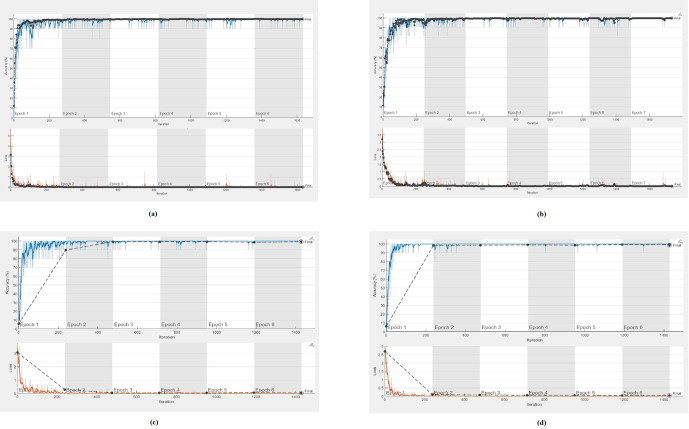
Accuracy and loss graphs of the classification process of the **(a)** AlexNet, **(b)** SqueezeNet, **(c)** GoogLeNet, and **(d)** Resnet101 models.

In this study, four different CNN models were experimented with to recognise buffalos from muzzle images. The first used model was AlexNet. For training, the samples were randomly split into training and validation sets. MiniBatchSize was selected to be 10, and the learning rate was set as 0.0001. SGDM was used as an optimiser. As a result, 851 of 852 samples were correctly recognised. The confusion matrix obtained from AlexNet is shown in Table 4, while accuracy and loss graphs are shown in Fig. 6a.

The second used model was SqueezeNet. For training, the samples were randomly split into training and validation sets. MiniBatchSize was selected to be 11, with a learning rate of 0.0002. SGDM was used as an optimiser. As a result, 851 of 852 samples were correctly recognised. The confusion matrix obtained from SqueezeNet is shown in Table 5, while accuracy and loss graphs are shown in Fig. 6b.

The third used model was GoogLeNet. For training, the samples were randomly split into training and validation sets. MiniBatchSize was selected to be 10, and the learning rate was set at 0.0003. SGDM was used as an optimiser. As a result, 851 of 852 samples were correctly recognised. The confusion matrix obtained from GoogLeNet is shown in Table 6, while accuracy and loss graphs are shown in Fig. 6c.

The fourth used model was ResNet101. For training, the samples were randomly split into training and validation sets. MiniBatchSize was selected to be 10, and the learning rate was set at 0.0003. SGDM was used as an optimiser. As a result, 846 of 852 samples were correctly recognised. The confusion matrix obtained from ResNet101 is shown in Table 7, while accuracy and loss graphs are shown in Fig. 6d.

The classification accuracy, precision, recall, and F1 score obtained with all four models are given in detail in Table 8.

**Table 8:** Classification accuracies, precision, recall, and F1 score of all models used in the study.

Model	Accuracy	Precision	Recall	F1 score
AlexNet	99.88 %	0.996	0.999	0.997
SqueezeNet	99.88 %	0.998	0.999	0.999
GoogLeNet	99.88 %	0.997	0.999	0.998
ResNet101	99.30 %	0.979	0.995	0.987

As seen from Tables 3–8 and Fig. 8, all four models were able to successfully recognise buffalo based on muzzle pattern. AlexNet, SqueezeNet, and GoogLeNet identified 851 images from 852, while ResNet101 was a bit unsuccessful in comparing them, with six misidentified images.

## 4 Conclusion

This study investigated buffaloes' recognisability using a buffalo muzzle pattern. The original dataset created within the scope of the study has been added to the literature. A recognition task was performed with a total of 11 buffaloes. Four CNN algorithms are employed, and their performance is evaluated. As a result, AlexNet, SqueezeNet, and GoogLeNet achieved 99.88 % classification accuracy, while SqueezeNet achieved the best F1 score. ResNet101 reached the worst result among the investigated algorithms with 99.30 % accuracy, which is a sufficiently successful result for this field. When the literature is examined, there is only one study on buffalo identification based on muzzle print. Singh et al. (2025) used the muzzle images of 198 Surti buffaloes. They applied four deep learning algorithms and achieved 90.8 % accuracy with the AlexNet algorithm. However, there are several studies on cattle identification based on muzzle print. The lowest accuracy was achieved by El-Henawy et al. (2016) at 89.64 % for 52 bovines. Shojaeipour et al. (2021) used the largest number of cattle. They achieved 99.11 % accuracy using YOLOv3–ResNet50. El Hadad et al. (2015) achieved 100 % accuracy using the kNN algorithm on 28 bovines. The other studies achieved approximately 98 % accuracy (Barry et al., 2007; Gaber et al., 2016; Kumar et al., 2018). As highlighted in the literature review, this study achieved successful results. The key contributions of this study include the creation of a buffalo image dataset, the experimentation with CNN models on livestock muzzle image patterns, and the evaluation of the results. In future research, a mobile application could be developed for this purpose. The study's major contributions include the creation of a unique buffalo muzzle image dataset, the application of CNN models for livestock muzzle pattern recognition, and the comprehensive evaluation of these models. These findings demonstrate the potential of CNN-based recognition systems in the livestock sector.

This research has practical implications for livestock management. Such recognition systems can offer significant advantages, including enhancing livestock tracking, reducing human error, and streamlining herd management processes. For farms, the adoption of these systems may provide economic benefits by optimising resource allocation and improving operational efficiency. Additionally, the potential development of a mobile application for this purpose could further enhance accessibility and usability in real-world scenarios. Future studies may focus on the economic feasibility of implementing these technologies at a larger scale, along with investigating their broader contributions to improving productivity and sustainability in livestock farming.

## Data Availability

The corresponding author can provide all raw data upon request.
